# How good is a PCR efficiency estimate: Recommendations for precise and robust qPCR efficiency assessments

**DOI:** 10.1016/j.bdq.2015.01.005

**Published:** 2015-03-11

**Authors:** David Svec, Ales Tichopad, Vendula Novosadova, Michael W. Pfaffl, Mikael Kubista

**Affiliations:** a Institute of Biotechnology, Academy of Science of the Czech Republic, Prague, Czech Republic; b Faculty of Medicine Pilsen, Charles University, Pilsen, Czech Republic; c TATAA Biocenter, Gothenburg, Sweden; d Physiology Weihenstephan, TUM – Technische Universität München, Freising, Germany

**Keywords:** RT-qPCR, reverse transcription-quantitative polymerase chain reaction, ANCOVA, analysis of covariance, E, PCR efficiency, Cq, cycle of quantification, GMO, genetically modified organism, ISO, International Organization for Standardization, IEC, International Electrotechnical Commission, RIN, RNA Integrity Number, NTC, no template control, FDA, food and Drug Administration, EPA, Environmental protection agency, CLSI, Clinical and Laboratory Standards Institute, MIQE, minimum information for publication of quantitative real-time PCR experiments, Real-time quantitative PCR, qPCR, Amplification efficiency, Standard curve, Dilution series, qPCR assay validation

## Abstract

We have examined the imprecision in the estimation of PCR efficiency by means of standard curves based on strategic experimental design with large number of technical replicates. In particular, how robust this estimation is in terms of a commonly varying factors: the instrument used, the number of technical replicates performed and the effect of the volume transferred throughout the dilution series. We used six different qPCR instruments, we performed 1–16 qPCR replicates per concentration and we tested 2–10 μl volume of analyte transferred, respectively. We find that the estimated PCR efficiency varies significantly across different instruments. Using a Monte Carlo approach, we find the uncertainty in the PCR efficiency estimation may be as large as 42.5% (95% CI) if standard curve with only one qPCR replicate is used in 16 different plates. Based on our investigation we propose recommendations for the precise estimation of PCR efficiency: (1) one robust standard curve with at least 3–4 qPCR replicates at each concentration shall be generated, (2) the efficiency is instrument dependent, but reproducibly stable on one platform, and (3) using a larger volume when constructing serial dilution series reduces sampling error and enables calibration across a wider dynamic range.

## 1 Introduction

Literature search (e.g. Pubmed) for scientific publications using the keyword “quantitative PCR” (qPCR) retrieves hundreds of thousands of hits, manifesting that qPCR has become mainstream life sciences technology [1], [2], [3]. It is widely acknowledged as the most sensitive method to quantify minute amounts of nucleic acids and its applications split into two main types referred to as: relative [4], [5] and absolute [6], [7], [8] quantification. In relative quantification the analyte, often reverse-transcribed mRNA or microRNA, is quantified relative to an endogenous reference [4], [5]. In absolute quantification the targeted nucleic acid (the analyte) is measured relative to a set of standards used to construct a standard curve [6], [7], [8]. The established name “absolute quantification”, is rather confusing, since absolute value are never determined; a more appropriate name would be “calibration”, as the concentration of the field sample in fact is measured “relative” to the concentrations of the standard samples.

The standard curve is also used to assess the performance of qPCR assay by estimating its efficiency [9] and optionally also determining the assay dynamic range, limit of detection and limit of quantification. For the estimation of PCR efficiency the standard used to construct the standard curve does not have to be calibrated. The efficiency (*E*) of PCR is defined as the fraction of target molecules that are copied in one PCR cycle [10], [11]. A properly designed assay shall, in the absence of interfering substances in the sample matrix, amplify target DNA with at least 90% efficiency [12], [13]. However, the experimental determination of PCR efficiency has been subject of many discussions, resulting even in some very inappropriate recommendations, such as to perform separate standard curves with few data points in every qPCR run to account for inter run variation. There is also a qPCR community that focuses on alternative procedures estimate PCR efficiency based on the analysis of individual amplification curves [14], [15]. In spite of these heroic efforts, the standard curve remains the most reliable and robust approach to estimate PCR assay efficiency that is broadly accepted by the community [16], while some of the alternative approaches have found use as quality control tools in high-throughput setups [17], [18], [19].

The estimation of PCR efficiency by means of a standard curve involves generating a series of samples with controlled relative amounts of targeted template. These samples are usually constructed by serial dilution of a concentrated stock solution, most frequently using 10-fold dilution steps. The so prepared standard samples are analyzed by qPCR measuring the quantification cycle (Cq) using standard procedures. A plot of the Cq's versus the logarithm of the target concentrations is constructed and is expected to be linear with a negative slope. For a 10-fold dilution series the slope is −3.33 when *E* = 100%. This follows from the assumption of a perfect doubling of the number of DNA template molecules in each step of the PCR (Eq. (1)).

(1) $$N_{x}=N_{0}2^{x}$$

Where *N*_*x*_ is the number of target molecules after *x* cycles and *N*_0_ is the initial number of double stranded target molecules. If the initial template is single stranded, such as cDNA, the first PCR cycle produces its complement rather than doubling it (Eq. (2)).

(2) $$N_{x}=N_{0}2^{\left(x-1\right)}$$

In practice, perfect doubling of the number of molecules in every cycle is highly uncommon; rather a fraction only is copied, which is the PCR efficiency (*E*). Hence, *E* is a number expected to be between 0 and 1 and is frequently expressed as percentage (Eq. (3)).

(3) $$N_{x}=N_{0}{\left(1+E\right)}^{\left(x-1\right)}$$

For example, let say a test tube contains 100 target molecules and after one amplification cycle it contains 180 molecules, *E* = 80%, since 80% of the target molecules present were amplified. In practice we do not measure the number of amplicons; rather we measure the fluorescence (*I*) from dyes or probes present in the reaction mix that bind to the amplicons formed (Eq. (4)).

(4) $$I={k^{\prime }}_{\left(\text{assay}\_1\right)}\times N_{x}$$

The fluorescence (*I*) depends on the amount of amplicon formed (*N*_*x*_) and $k^{\prime }$ is a proportionality constant. It reflects the amount of fluorescence produced per amplicon formed, and may change during the course of the reaction as the reporter/DNA ratio changes. The thermodynamics behind is complex, although some brave attempts to model it have been made [20]. Modelling the thermodynamics is, however, not needed in order to compare samples at a fluorescence threshold (*I*_threshold_), which is the normal practice to analyze qPCR data, since at threshold all reactions based on the same assay contain the same number of amplicons (Eq. (5)):

(5) $$I_{\text{threshold}}=k^{\prime }\times N_{0}{\left(1+E\right)}^{\left(\text{Cq}-1\right)}$$

and the effect of $k^{\prime }$ cancels. Rearranging Eq. (5) produces the relation between PCR efficiency and the slope of the standard curve found in textbooks (Eq. (6)) [2], [21].

(6) $$E=10^{-(1/\text{slope})}-1$$

The PCR efficiency depends on many factors including: (1) the assay performance, which depends on the primers’ and template sequences and structures. Secondary structure and opportunity for undesired intra-molecular interactions reduce PCR efficiency; (2) the sample matrix, which may contain inhibitors and other interfering substances from the sample or carry overs agents from upstream processing steps; (3) reagents used and their concentrations. Essentially, any of the PCR reagents can be rate and performance limiting [22] including PCR protocol; and (4) competing reactions.

The samples shall be tested for inhibition, which is easy done using RNA or DNA spikes [23], [24]. It can also be observed by performing a serial dilution [25]. In fact inhibition is often the cause of unrealistic PCR efficiency estimates (*E* > 100%) as it is pronounced in the most concentrated samples leading to deviation from linearity. If ignored and mistakenly included in the linear fit, those samples reduce the slope leading to too high PCR efficiency estimates. In some cases inhibition is pronounced only in the upstream reactions such as the reverse transcription and not noticed in qPCR.

A template must be chosen for the assessment as well as the matrix. Choosing a matrix characteristic of the field sample the estimated efficiency will reflect the performance of the PCR assay in the actual samples that will be analyzed. This, however, requires pure matrix is available. Usually a new assay is first validated in a pure matrix devoid of interfering agents. Assays that show high PCR efficiency are robust and will be less prone to inhibition in complex matrices. Purified PCR product is often used as template for PCR efficiency estimates, because it is easy to produce. However, it often leads to side reactions because of its short length, and it does not reflect the effect of flanking sequences that may interfere with PCR by wrapping onto the template [26]. Such interference can be significant in the initial cycles of the PCR, when the original template is abundant, and influence the measured Cq. For validation of assays for gene expression profiling a cDNA library is a suitable source of long template molecules with representative secondary structures. Genomic DNA or plasmids containing the gene of interest can be used as standard for validation of assays for DNA analysis, preferably after excising a fragment containing the target sequence to remove interfering supercoiling [26]. Still another option is to use synthetic templates (e.g. gBlocks – IDT, GeneArt – LT).

**Fig. 1:**
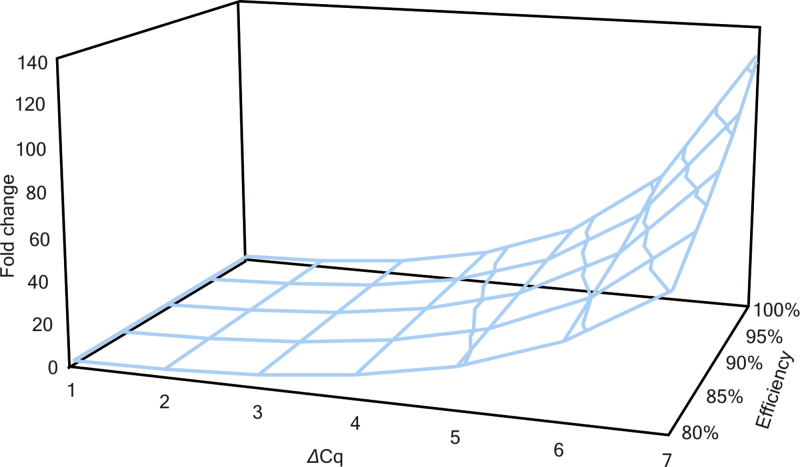
Impact of PCR efficiency on fold change between two conditions. Model shows how determination of efficiency impacts relative fold change calculated in terms of difference in Cq values (ΔCq).

The performance of new assays needs to be tested by means of specificity, efficiency and sensitivity (sometimes also for limits of detection and quantification). While properties of a good qPCR assay are well described by means of specificity in MIQE guidelines [16], where tests and optimal criteria are recommended, e.g. in silico BLAST (single unique complementarity), electrophoresis (single band of correct size), melt curve (single peak in target amplification, no peaks in NTC while Cq of NTC ≤40 can be ignored if ΔCq of NTC and target is ≤5), “no RT” control (ΔCq of no_RT and RT ≤5). Detailed piece of information about what parameters to use for optimal efficiency estimate using standard curves was missing and our work offers detailed evidence. The PCR efficiency is one of the most important indicator of the performance of a qPCR assay and is also required parameter for quantitative analysis when fold changes are calculated. Proper usage of PCR efficiency in qPCR analysis requires it is estimated with high precision. Inaccurate estimations of *E*_*x*_ can lead to substantial under- or overestimation of the calculated fold change, particularly when large differences in expression are measured (Fig. 1).

The aim of this study is to test the impact of three experimental factors on the precision of the estimated PCR efficiency: (1) the effect if qPCR instrument changes; (2) the effect of how many technical replicates are included; and (3) the effect of the volume transferred across dilutions.

## 2 Results

We tested three experimental factors in terms of precision of the estimated PCR efficiency: (1) the effect of the qPCR instrument; (2) the impact of the number of technical replicates; and (3) the effect of the volume transferred across dilutions. In all cases we postulate the null hypothesis as there is no difference to be observed due to these three factors.

### 2.1 The effect of the qPCR instrument

We performed one dilution series consisting of 10-fold dilution of 6 concentrations, each was analyzed using 4 qPCR replicates (6 × 4) on 6 different qPCR instruments: Eppendorf RealPlex, BioRad CFX96, AB StepOne, AB 7500Fast, Corbett Rotorgene I, Roche LC480. Three highly abundant rRNA (18S) and mRNAs (Hprt, H2afz) were used to minimize the effect of sampling error.

The slopes of the standard curves were compared with Analysis of covariance (ANCOVA) using a model based on the three terms (i), (ii) and (iii) which were tested simultaneously using the SS II type of error. Sum of squares were calculated and the *p*-values associated with the *F*-distribution were obtained.

(i) The impact of instrument on the standard curve intercept was very significant (*p* < 0.0001). This was expected, since the Cq-values and, hence, the intercept depends on instrument settings (gain, threshold) as well as instrument factors, such as excitation and emission details (wavelengths, slit widths, filters, exposure time, etc.).
(ii) The effect of log_10_(dilution) with *p* < 0.0001 is what describes the slope of the curve and the expected growing Cq value in response to decreasing concentration, in other words the qPCR efficiency.
(iii) The interaction between the instrument and the slope is the relevant test result. It reflects correlation of the instrument used and the slope of the standard curve, which in turn determines the PCR efficiency. Hence, this reflects the influence of the qPCR instrument on the measured PCR efficiency. Test result was very significant (*p* < 0.0001).

**Fig. 2:**
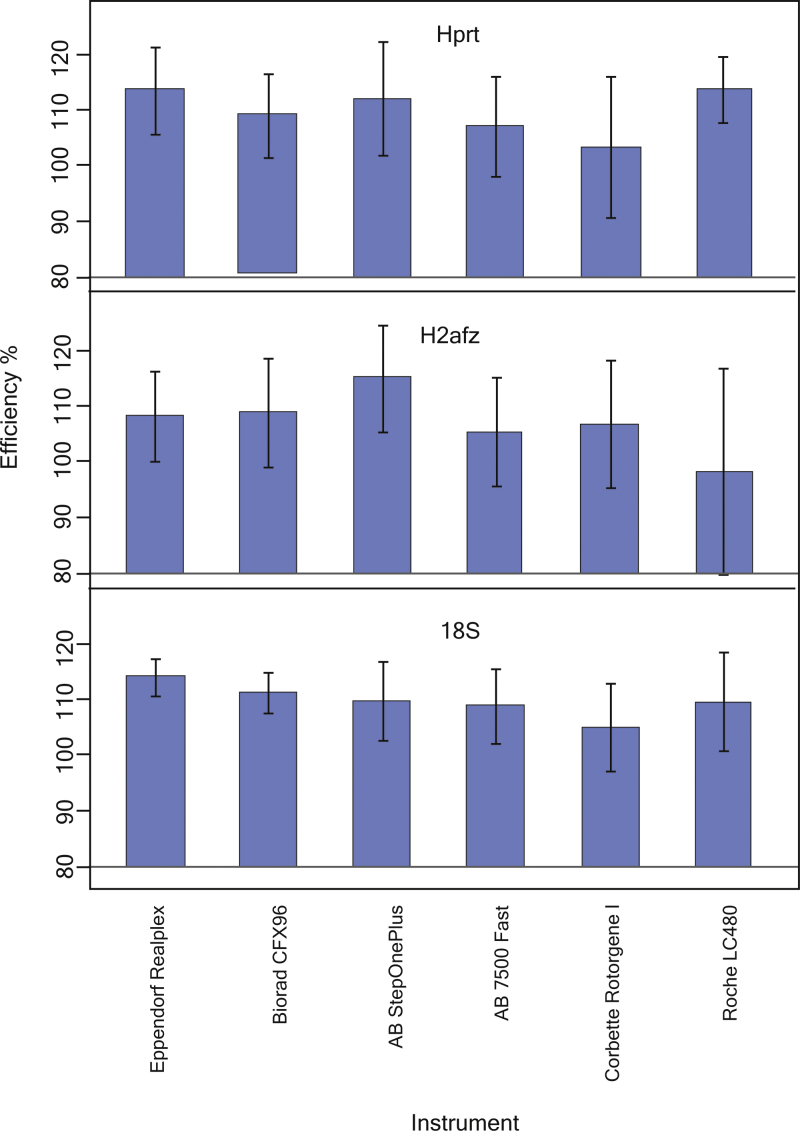
PCR efficiency estimated for three selected assays using six different instruments. Error bars indicate 95% confidence interval. Efficiencies were estimated from standard curves based on ten-fold dilutions based on six concentrations, each analyzed in four qPCR replicates.

Our results show that the qPCR instrument used has an impact on the efficiency estimate (Fig. 2). Hence, the same assay in the same matrix experiences different PCR efficiency in different instruments. Most likely this is due to instrument specific hardware properties and settings, software settings and the particular algorithm used to extract the Cq values.

The objective of this study was not to compare instruments in order to find a best one, since performance may depend on the reaction conditions chosen, but to test if there is an effect of instrument. Results are clear; there is significant effect. The instrument specific properties conceived to affect the PCR efficiency are the reaction container, which here in most cases were plastic microtiter well plates, that influences the rate and efficiency of heat transfer, the heating and cooling capacities, which may induce thermal over- and undershoots, the thermal unit and its control of the actual temperatures in the reaction mix (instruments generally register the temperature of the heating block, which may differ from the temperature of the reaction mix), and the heating and cooling rates.

### 2.2 The effect of technical replicates

We collected two data sets to test the impact of technical replicates on the precision of the estimated PCR efficiency. In the first data set we used 6 dilution steps that each was measured in 4 qPCR replicates for 18S. To test the influence of replicates the data were analyzed as follows. We performed more than 1000 samplings of the data randomly selecting one, two or three out of the four technical replicates at each concentration, and compared these to evaluate the robustness of the standard curve method to estimate PCR efficiency. With samplings we tested the majority of all possible combinations and accurately modelled the imprecision of the estimation of *E*. The impression, measured as standard deviation (SD), of *E* depends on the number of technical replicates at each concentration (Supplement Table 1). Employing a permutation test with 3 replicates we obtained 0.934 < *E* < 0.966, SD = 0.007. With 2 replicates 0.923 < *E* < 0.980, SD = 0.013, while with a single measurement at each concentration we obtained 0.894 < *E* < 0.991, SD = 0.022. The impression, expressed as confidence interval, ranges from 8.3% without replicates to 2.3% when performing triplicates. Best estimate of *E* is expected with all four technical replicates. This gave *E* = 0.951 (Supplement Table 1). SD cannot be estimated for four replicates by resampling, since only a single sampling is available, but it can be estimated from the error of the slope (Eq. (7)):

(7) $$\text{SE}(E)=\text{SE}(\text{slope})\times \frac{(1+E)\;\ln\;10}{{\text{slope}}^{2}}$$

This gives SE(*E*) = 0.008. From SE(*E*) the 95% confidence interval is calculated as Eq. (8).

(8) $${\bar{E}}_{t}\pm t_{95\%,n-2}\times \text{SE}({\bar{E}}_{t})$$

and was 2.6% as no selection of “best” datapoints was possible, compared to triplicate assessment.

Supplementary Table 1 related to this article can be found, in the online version, at doi:10.1016/j.bdq.2015.01.005.

Supplement Table 1 Measured Cq values used in the first data set in the study.

**Fig. 3:**
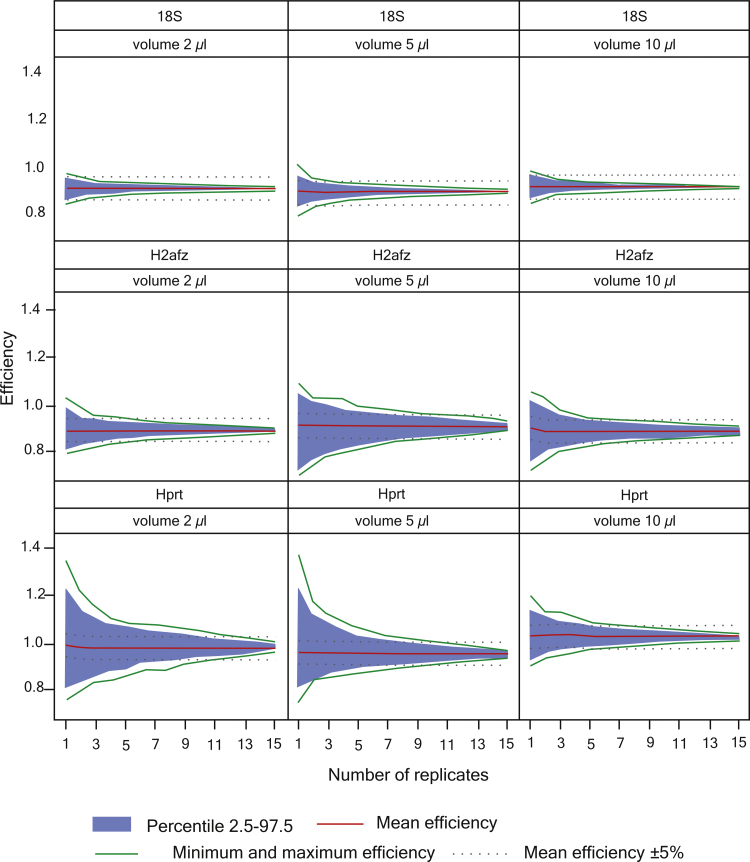
Effect of transfer volume when constructing standards by means of serial dilution. Four dilution series were constructed using each of the transfer volumes 2 μl, 5 μl and 10 μl. Each standard curve was based on ten-fold dilutions for 6 concentrations, each analyzed in four qPCR replicates. This gave a total of 16 qPCR measurements at each dilution step for each transfer volume tested, and were subject to Monte Carlo analysis. Missing data were not replaced or imputed in the analysis.

The second data set was also based on six 6 concentrations, each analyzed in 16 qPCR replicates. Also with this set the accuracy of the efficiency estimate increased with the number of replicates. For the highly abundant 18S ribosomal RNA the confidence interval decreased from 12.4% to 0.8% when increasing the number of qPCR replicates from one to 15 replicates. For mRNAs the uncertainty dropped from 32.0% to 3.1% (H2afz) and 42.5% to 3.3% (Hprt) when increasing from one to 15 qPCR replicates (Fig. 3). This comparison suggest the precision of PCR efficiency estimate also depends on the concentration of the target RNA, or strictly on the target cDNA produced, due to sampling error, which becomes pronounced in the most diluted steps [27], [28]. This may be a problem when total RNA or cDNA library is used to produce standards by sequential dilution for efficiency estimations of new assays and the concentrations of target transcripts are unknown. We therefore also tested the effect of the volume transferred when constructing the standards by serial dilution.

### 2.3 The impact of transfer volume in serial dilutions

**Fig. 4:**
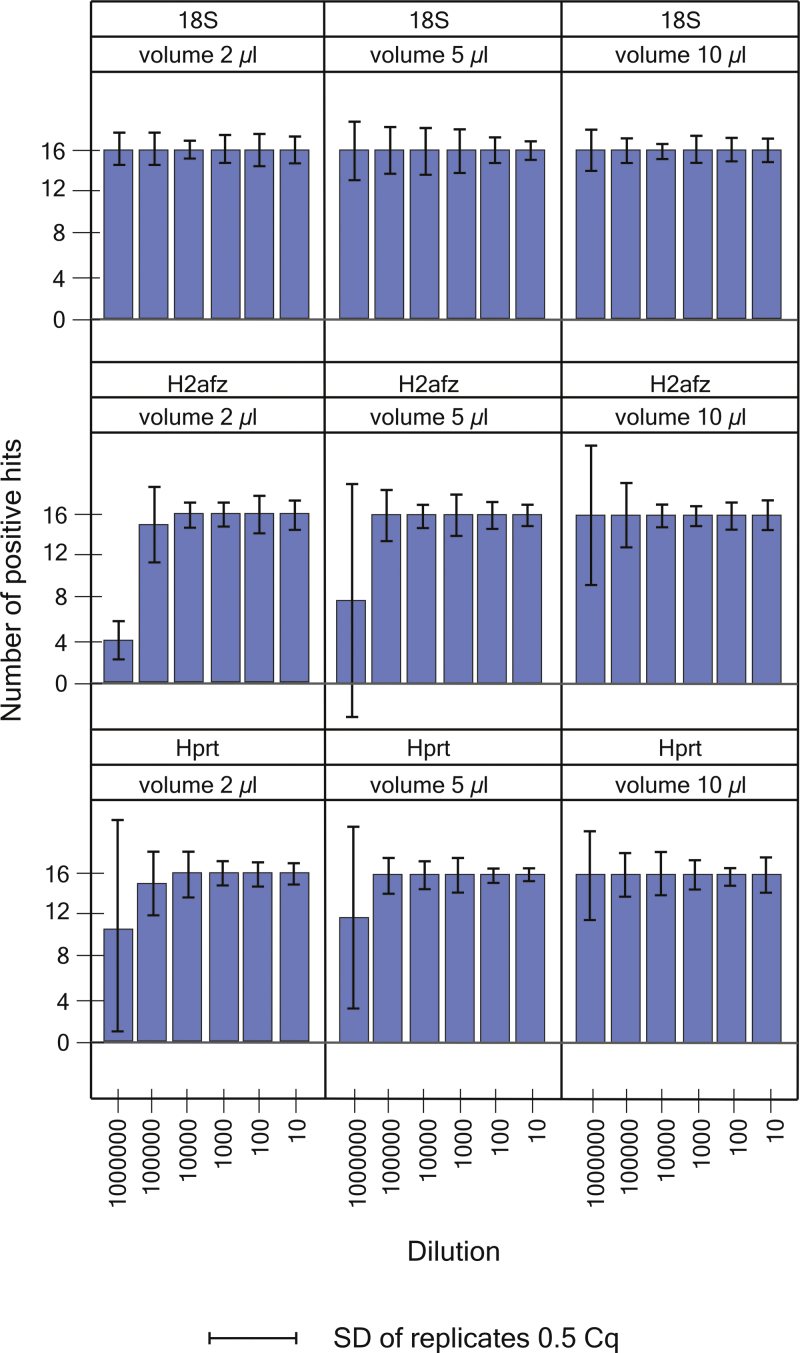
Effect of the transfer volume used in the serial dilution to construct the standards on the number of positive amplification reactions (hits) and on the repeatability of technical replicates at the most diluted standard. Transfer volumes were 10 μl, 5 μl and 2 μl. Four dilution series were performed for each transfer volume and each assay, based on 6 concentrations and analyzed in qPCR tetraplicates, resulting in 16 qPCR datapoints per concentration per assay per volume.

To test the impact of transfer volume when constructing standard samples by serial dilution of a concentrated stock on the imprecision of the PCR efficiency estimate, we constructed standards using transfer volumes of 10 μl, 5 μl and 2 μl. Four independent sets of standards were constructed by serial dilution with each transfer volume. Each standard curve had 6 concentrations made by 10-fold dilution and the mRNA targets were analyzed at each concentration in 4 qPCR replicates. The diluted samples of all tested volumes should have therefore identical concentrations. In total, for each target and transfer volume we obtained a maximum of 16 Cq values at each concentration. We selected 18S rRNA to represent a target with high-abundancy over the whole dilution series, while Hprt and H2afz represent more “real life” transcripts, which may reach the level of very few copies within the dilution series. If very few copies are present per sample, rapid increase of sampling error and loss of data are observed and usually make the efficiency estimate less reliable. For each target a total of 288 (3 transfer volumes × 4 standard curves × 6 concentrations × 4 replicates) qPCR measurements were performed using 384 block of LC480. Some of qPCRs, in the most extensively diluted standards for the least abundant genes did not yield Cq values. The data were analyzed by comparing the frequency of missing data for each target at each concentration and the SD of the qPCR replicates (Fig. 4). For the abundant 18S rRNA no dependence of transfer volume on the precision of the PCR efficiency estimate was observed, presumably because its concentration never reaches below the 30–35 copies per transferred volume, where sampling error due to Poisson distribution becomes significant [29]. For Hprt mRNA SD of qPCR replicates and the frequency of missing at the most diluted sample increased with lower transfer volume. When using 2 μl transfer volume 31.25% of the replicates at the most diluted standard were missing and SD = 2.05 cycles. For 5 μl transfer volume frequency of missing data at lowest concentration was 25.0% and SD = 1.74 cycles. For 10 μl transfer volume no data were missing and SD = 0.85 cycles. H2afz transcripts were even less abundant. Important sampling error was introduced and the frequency of missing data in the most diluted sample was large: frequency missing data was 68.75% and SD = 0.35 cycles with 2 μl transfer volume; 50% missing data and SD = 2.25 cycles with 5 μl transfer volume; and with 10 μl transfer volume there were no missing data and SD = 1.34 cycles. Missing data is a problem when assessing PCR efficiency by means of a standard curve. Occasionally missing data are ignored by the operator, which is wrong, since such action reduces the estimated SD giving the imprecision data are of high quality, when, on the contrary, there are reproducibility issues. Rather, the dynamic range of that particular assay is not wide enough to cover the most diluted standard. The dynamic range is based on estimating the assay limit of quantification (LoQ), which is based on the SD's of replicates and can be done, e.g. with the GenEx software (MultiD Analysis). In our example, we show using larger transfer volume the precision of the estimated PCR efficiency is improved and the assay dynamic range is widened for the H2afz transcript.

## 3 Discussion

Validation and standardization of qPCR measurements [16], [30] and RNA processing [31] is receiving increasing attention from regulatory bodies such as the US Food and Drug Administration (FDA) [32], the Environment Protection Agency (EPA) [33], and standardization organizations such as the Clinical and Laboratory Standards Institute (CLSI, earlier called NCCLS) [34] and most recently the European Committee for Standardization (www.cen.eu) and the International Organization for Standardization (www.iso.org), as well as multinational projects (www.spidia.eu).

Assessing PCR efficiency by means of a standard curve is a quality benchmark for the assay as well as for the pre-analytical process, if tested in representative sample matrix and is generally recommended in the MIQE guidelines [16]. The estimated PCR efficiency also enter calculation of gene expression regulatory levels [4], [5]. Although the qPCR is a widely used technique, recommendations on how to use a standard curve to estimate PCR efficiency are rare and a general consensus or guidelines are lacking. It is known that estimates of PCR efficiency may be influenced by several experimental factors. Although there is a widely elaborated statistical theory [35], [36] as well as recommendations by international organizations for clinical applications that include use of the calibration curve [37], the PCR efficiency estimation requires special attention as basic principles differ from the common biochemical and analytical procedures. The unconventional exponential character of PCR (that can, however, be linearized by applying log transform) requires a rather complex set-up and statistical analysis.

Using a Monte Carlo approach we tested how the number of replicates used in the standard curve influences the precision of the PCR efficiency estimate. We also tested how robust the PCR efficiency is, considering the random effect contributed by the qPCR cycler. With our first data set we found the precision increased by 41% when using two replicates instead of a single measurement at each concentration, and by 68% when using three replicates. These observations were confirmed using a second data set with 16 replicates at each concentration. In the second set we also varied the transfer volume in the serial dilution used to construct the standards and experimentally demonstrated that larger transfer volumes reduce sampling error improving precision and widening the dynamic range of the assay.

In a statistical test, when the tested factor – here the log_10_ dilution on the Cq – might show heterogeneity, analysis of covariance provides an adequate statistical ground to test the null hypothesis of homogeneity by testing for interaction between the presumed heterogeneity factor and the tested factor. This can be visualized in a bar plot, similar to Fig. 2, where variation in height indicates interaction. In our study we tested the effect of different instruments, which was profound. Similarly the effects of plastic ware, plate sealing strategy, extraction kit, transportation, storage means, RT and PCR chemistries, experimental protocols as well as reagent batches, lots and even suppliers for presumably equivalent products (i.e. oligonucleotide suppliers) can be tested.

We show in our study that PCR efficiency can be estimated with established statistical theory [35] in line with existing laboratory guidelines [37], [38]. Based on our findings and in accord with the CLSI guidelines, mainly the EP6-A guideline [37], [38] for the estimation of PCR efficiency (*E*) by means of a qPCR standard curve we recommend:

• The standard curve used to establish amplification efficiency may be different from the standard curve used to estimate target quantities in unknown field samples. The standard curve to assess PCR efficiency can be based on a clean matrix to assess the performance of the PCR assay in absence of interference. The standard curve used to estimate concentrations of field samples must be based on representative field matrix to account for inhibition and interference in the field samples.
• The concentration range used to establish amplification efficiency should cover the range of the anticipated experiment or routine application and preferably expand beyond by at least 20%, since predictive precision at the extreme concentrations of a standard curve are always compromised. Too extensive standard curves should be avoided, since measurements at extreme concentrations may deviate from linearity and give inaccurate estimate of the amplification efficiency. This is true at high as well as at low target concentrations. Very high target concentration can have such low Cq value that base-line subtraction becomes problematic. Indeed, deviations at high target concentration due to base-line subtraction problems are likely cause of occasional reports of PCR efficiencies far exceeding the theoretical maximum of 100%. To avoid such errors a qPCR standard curve should be tested for linearity according to EP-6 [37] to determine its linear range (using e.g. GenEx – MultiD, SPSS). The analyte concentration does not have to be known to estimate the PCR efficiency. It is sufficient to know the relative concentrations between the standards, which are given by the dilution factor when preparing the standards by serial dilution.
• Generally serial dilutions to produce standard curves are discouraged because of carry-over of error [39]. In diagnostic applications, when a limited concentration range is of interest, standards can be produced by the mixing of a low and high concentrated stock. However, in most PCR applications assays shall cover a wide dynamic range, which requires serial dilution of a concentrated stock to produce the standard samples. It is then advisable to use as large transfer volume as possible across the dilution steps to minimize sampling and pipetting (DIN 12650, ISO 8655-2) errors. We recommend using at least 5 μl transfer volume and generally discourage pipetting volumes less than 2 μl using conventional pipettes.
• Five dilution steps, corresponding to 6 different concentrations if the stock is used as a standard, is minimum to identify the assay linear range and reliably estimate *E*. CLSI guidelines [37] recommended that five to seven concentrations in at least two dilution series should be used when a linear performance within a routinely anticipated range is to be proven. Developers of new methods who want to establish linear range should use minimum of seven to eleven concentrations covering the range of interest, with each concentration represented by at least two replicate. The concentrations should extend over the anticipated range by at least 20%.
• If standard concentrations in log scale are evenly spread an even number of concentrations is preferred due to the heaver effect.
• When used for calibration the standard curve should be determined for a standardized procedure based on a particular extraction kit, RT kit, one type of instrument, a particularly microtiter plate and sealing strategy, and particular batches of reagents. If there is any change in protocol, a new reagent batch is used, consumables are replaced or instrument is changed new standard curve shall be collected.
• When used for calibration the standard samples shall be as similar as possible to the field samples to appropriately reflect the impact of the sample matrix. When experiment with many biological subjects is planned, pooled samples can be used to construct the standards. If different types of samples are analyzed, for example serum, plasma, urine separate standard curves shall be constructed for each sample type.
• It is not recommended to perform separate estimates of *E* in each experiment run and use it to correct for variation between runs, since this may potentially introduce large imprecision and even systematic bias into the measurements that are larger than the inter-run effect compromising data quality rather than improving it. It is particularly inadvisable to perform minimal standard curves based on few concentrations only in each run. Such standard curves will report different PCR efficiency in each run, but this variation is due to random effects in each run rather than reflecting true systematic differences. Better is to perform a single, highly precise estimation of the PCR efficiency at conditions representative of the study that is used for all calculations, and correct variation between runs using a robust inter-plate calibrator instead measured at least in triplicate (e.g. TATAA Biocenter) [40].
• When designing standard curves for calibration the entire testing process shall be covered, including the pre-analytical process comprising extraction, storage and purification steps and reverse transcription in the case of RNA analysis.

Factors that may potentially influence the experiment and thus the standard curve and PCR efficiency are the laboratory, operator, chemistry, instruments, plastics, minor changes in protocol, etc. When they have to be varied, for example, when used in large laboratories, in inter-laboratory exercises or when products are developed for use on multiple platforms at customer laboratories, the influence of these factors shall be established by means of inferential statistics. The procedure we used here to test the influence of instrument is appropriate. Standard curves are constructed and confidence intervals of the slopes are established, which allows testing the effect of interaction between the tested factor and the log_10_ of the concentration on Cq by means of analysis of covariance.

## 4 Methods

We designed a one factor test of robustness of the estimation of PCR efficiency on different instruments within the qPCR core facility of the Institute of Biotechnology, Prague. Further, we tested the effect of replicate design on the imprecision of the estimated *E*. Following routine practice, the standard material stock was prepared only once and used as starting material in all experiments. Reverse transcribed 18S rRNA and the mRNAs Hprt and H2afz were used as representative targets. When assessing the sampling error in sequential dilution using different transfer volumes the same cDNA stock was used as starting material to construct independent dilution series.

### 4.1 Extraction of RNA and synthesis of cDNA

The cDNA was prepared from murine total RNA isolated separately from liver, spleen, brain, and muscle that was pooled for RT. Organs were homogenized in Trizol (Invitrogen) using Ultra-turrax T25 (IKA Labortechnik) immediately after collection and stored at −80 °C. The isolation of total RNA was performed according to manufacturer's instructions without DNase treatment. Total RNA dissolved in RNase free water, stored in −80 °C at a concentration of 2.9 μg/μl (Nanodrop) with RNA Integrity Number (RIN) number of 9.5 (Agilent Bioanalyzer) was used for reverse transcription (RT). SuperScript III RT kit (Invitrogen) was used to reverse transcribe RNA into cDNA in 50 μl, containing 13.05 μg of total RNA, 0.5 mM of dNTP (Promega), 2.5 mM of oligo dT (18 bp, Eastport) and 2.5 mM of random hexamers (Eastport) following manufacturer's instructions. After RT all samples were diluted to 500 μl with nuclease free water. All assay showed ΔCq larger than 10 between RT and RT(−) control evidencing negligible DNA background. Inhibition in the RT-qPCR workflow was tested using an RNA spike (TATAA Biocenter) and no inhibition was detected.

### 4.2 Preparation of standard curves

A pipetting robot EP Motion EP5070 (Eppendorf) was used to construct all dilution series and to set up all qPCR plates. Each cDNA dilution series was based on six cDNA standard samples prepared by five steps of sequential 10-fold dilutions.

In the first data set each cDNA standard was analyzed in four qPCR replicates generating a total of 24 (6 × 4) Cq values. The following liquid handling protocol was used: 550 μl of diluted cDNA was dispensed in Standard 1 (St1), 450 μl of nuclease-free water containing carrier 50 ng/μl LPA (GeneElute, Sigma Aldrich) was dispensed into standards St2 to St6. After robotic mixing (3 cycles of influx/release of 50% volume) of St1, 50 μl was transferred into St2. New pipette tip was used for mixing of St2 and the same procedure was performed for each subsequent standards. The same prepared standards and mastermix premixed with primers for each assay was used for all qPCR instruments. During dispensing (approx. 2 h) low temperature was maintained using pre-cooled metal blocks. The liquid handling was performed in a dark room to avoid exposure to light.

Dilution standards for the second data set were prepared similarly: the transferred cDNA volumes were 2 μl; 5 μl and 10 μl and were pipetted into 18 μl; 45 μl and 90 μl of nuclease free water containing carrier 50 ng/μl LPA (GeneElute, Sigma Aldrich), respectively. For each transfer volume, four independent dilution series were performed.

### 4.3 qPCR amplification

We selected mouse 18S (refseq NR_003278.1), H2afz (NM_016750.3) and Hprt (NM_013556.2) for our study because of its high abundance in various tissues making it possible to cover a wide dynamic range. The following primers were used to amplify 80 bp of the 18S sequence: Fwd 5-GAGAAACGGCTACCACATCC-3, Rev 5-TTTTTCGTCACTACCTCCCC-3; 133 bp amplicon of H2afz: Fwd 5-ACAGCGCAGCCATCCTGGAGTA-3, Rev 5-TTCCCGATCAGCGATTTGTGGA-3; 115 bp amplicon of Hprt: Fwd 5-GCTTGCTGGTGAAAAGGACCT-3, Rev 5-CTGAAGTACTCATTATAGTCAAGGGCA-3. qPCR reaction volumes were 20 μl containing iQ SYBR Green Supermix (Bio-Rad) and a final concentration of 400 nM of each primer (Eastport). For the plating of the first data set the EP motion 5070 (Eppendorf) robot initially dispensed 15 μl of mastermix containing primers (multidispense mode) and then added 5 μl of diluted cDNA (pipette mode). For the second data set the robot first dispensed 18 μl of mastermix and then added 2 μl of cDNA. All primers were designed with Primer3 (http://frodo.wi.mit.edu/primer3/), where we used mostly default parameters, except: amplicon size range was set to 60–150 bp to minimize complexity of PCR, increase efficiency and to facilitate use of the assay on fragmented material; Max. 3′ stability was set to minimum to reduce primer–dimer formations; optimum Tm was set to 60 °C, however optimal annealing temperature was validated using gradient qPCR (CFX 96, Bio-Rad) to maintain high efficiency. Temperature profile used was: 95 °C for 3 min followed by 40 cycles of amplification (95 °C for 20s, 58 °C for 20 s and 72 °C for 20 s). Assays were validated using identical qPCR protocol on duplicate non template controls (NTC), 2 RT minus (RT−) controls and 5-step 5-fold dilution series with four qPCR replicates per step with at least 90% efficiency (LightCycler 480, Roche Diagnostics, Germany). The formation of expected PCR products was confirmed by melting curve analysis and agarose gel electrophoresis. All PCR products showed single peak melting curve and uncompromised specificity.

### 4.4 Robustness test: the effect of instrument

As example of a robustness test of relevance in daily routine in many qPCR laboratories the influence of qPCR instrument was investigated. One serial dilution was used to generate 6 standard samples that each was measured in qPCR tetraplicates on 6 different plates (using recommended plastic for each instrument) on 6 different instruments. The instruments were: Bio-Rad CFX 96 (Bio-Rad), ABI 7500 Fast (Applied Biosystems), ABI StepOnePlus (Life Technologies, Applied Biosystems), RotorGene 6000 duplex (Qiagen), Realplex4 gradient S Mastercycler (Eppendorf), and 384 well plate platform LightCycler 480 (Roche Diagnostics). The same qPCR master mix stock was used in all runs. Linear regression was used to associate the measured Cq values with the log_10_ of the dilution factor (the true concentrations were not known). The stock concentration was arbitrarily assigned 10^6^× and each subsequent dilution was 10-times less. The Cq as response variable, the dilution factor and the instrument as the explanatory variables were used in a linear statistical model (Eq. (9)).

(9) $${\text{Cq}}_{ij}=\mu +\delta_{i}+\beta_{i}\;\log{\left(\pi \right)}_{ij}+e_{ij}$$

Where *δ* denotes the effect of instrument *i* on the intercept, the *β*_*i*_ log(*π*)_*ij*_ denotes the Cq_*i*_ response to the log of the dilution factor *π* for each dilution *j* for instrument *i* (interaction). *e* is the residual error within the block of replicates of sample dilution *j* and instrument *i*. The significance of the interaction term *β*_*i*_ log(*π*)_*ij*_ was tested using SAS v 9.1 for Windows, employing the GLM procedure as follows:

*proc glm data=>dataname<*;
*class instrument*;
model ct=instrument logconc logconc*instrument/solution noint ss2;
*run*;

This annotation delivers *p* values on tests based on the hypothesis of null effect of the instrument on the Cq intercept, the log of concentration on the Cq, and the interaction between the instrument and the log of the dilution factor on Cq.

### 4.5 The effect of technical replicates

Using a Monte Carlo approach we sampled over 1000 data subsets randomly selecting one, two, or three replicates out of each set of four performed at each concentration. This set contained the majority of the permutations possible and should adequately model the imprecision. The CI 95% taken as measure of the imprecision of *E*, depended strongly on the number of technical replicates performed at each concentration (Supplement Table 1). From the permutations the expectation value of *E* and its 95% confidence interval, taken as the samples with *E* in the range from the lower 2.5 and the upper 97.5 percentiles around the expectation value (2.5% < *E* < 97.5%), was calculated for each sampling with 1, 2 and 3 randomly selected technical replicates at each concentration.

### 4.6 The effect of volume transferred across the dilution series

Also the second data set with 16 replicates at each concentration was subjected to Monte Carlo analysis (Supplement Table 2) to obtained expectation values for *E* (Fig. 3, Fig. 4). The frequency of missing data at highest dilution and SD of the Cq's of the technical replicates were used as indicators of sampling error for the serial dilutions with transfer volumes of 2 μl, 5 μl and 10 μl. LC480 (Roche) with 384 block was used for generating second data set.

Supplementary Table 2 related to this article can be found, in the online version, at doi:10.1016/j.bdq.2015.01.005.

Supplement Table 2 Measured Cq values used in the second data set in the study.

## Author contributions

A. Tichopad, D. Svec, M. Kubista and MW. Pfaffl wrote the main manuscript, D. Svec performed the experiments, MIQE compliance and submission. Figures were prepared by V. Rusnakova. All authors reviewed the manuscript.

## Competing financial interests

The authors declare no competing financial interests.

## References

[1] Higuchi R. (1992). Simultaneous amplification and detection of specific DNA sequences. Biotechnology (N Y).

[2] Kubista M. (2006). The real-time polymerase chain reaction. Mol Aspects Med.

[3] Wittwer C.T. (1997). Continuous fluorescence monitoring of rapid cycle DNA amplification. Biotechniques.

[4] Hellemans J. (2007). qBase relative quantification framework and software for management and automated analysis of real-time quantitative PCR data. Genome Biol.

[5] Pfaffl M.W. (2001). A new mathematical model for relative quantification in real-time RT-PCR. Nucleic Acids Res.

[6] Boeuf P. (2005). CyProQuant-PCR: a real time RT-PCR technique for profiling human cytokines, based on external RNA standards, readily automatable for clinical use. BMC Immunol.

[7] Bustin S.A. (2000). Absolute quantification of mRNA using real-time reverse transcription polymerase chain reaction assays. J Mol Endocrinol.

[8] Larionov A., Krause A., Miller W. (2005). A standard curve based method for relative real time PCR data processing. BMC Bioinformatics.

[9] Stolovitzky G., Cecchi G. (1996). Efficiency of DNA replication in the polymerase chain reaction. Proc Natl Acad Sci U S A.

[10] Alvarez M.J. (2007). Model based analysis of real-time PCR data from DNA binding dye protocols. BMC Bioinformatics.

[11] Lalam N. (2006). Estimation of the reaction efficiency in polymerase chain reaction. J Theor Biol.

[12] Roche (2000). Overview of LightCycler quantification methods.

[13] AppliedBiosystems (2004). Amplification efficiency of TaqMan^®^ gene expression assays.

[14] Goll R. (2006). Evaluation of absolute quantitation by nonlinear regression in probe-based real-time PCR. BMC Bioinformatics.

[15] Tichopad A. (2003). Standardized determination of real-time PCR efficiency from a single reaction set-up. Nucleic Acids Res.

[16] Bustin S.A. (2009). The MIQE guidelines: minimum information for publication of quantitative real-time PCR experiments. Clin Chem.

[17] Bar T., Kubista M., Tichopad A. (2012). Validation of kinetics similarity in qPCR. Nucleic Acids Res.

[18] Sisti D. (2010). Shape based kinetic outlier detection in real-time PCR. BMC Bioinformatics.

[19] Tichopad A. (2010). Quality control for quantitative PCR based on amplification compatibility test. Methods.

[20] Gevertz J.L., Dunn S.M., Roth C.M. (2005). Mathematical model of real-time PCR kinetics. Biotechnol Bioeng.

[21] Mackay I.M. (2007). Real-time PCR in microbiology: from diagnosis to characterization.

[22] Raghavachari R., Tan W. (2001). Genomics and proteomics technologies.

[23] Nolan T. (2006). SPUD: a quantitative PCR assay for the detection of inhibitors in nucleic acid preparations. Anal Biochem.

[24] Svec D. (2013). Direct cell lysis for single-cell gene expression profiling. Front Oncol.

[25] Stahlberg A. (2003). Quantitative real-time PCR method for detection of B-lymphocyte monoclonality by comparison of kappa and lambda immunoglobulin light chain expression. Clin Chem.

[26] Pfaffl M., Bustin S.A. (2004). Quantification strategies in real-time PCR, A–Z of quantitative PCR.

[27] Good I.J. (1986). Some statistical applications of Poisson's work. Statistical science.

[28] Peccoud J., Jacob C. (1996). Theoretical uncertainty of measurements using quantitative polymerase chain reaction. Biophys J.

[29] Bengtsson M. (2008). Quantification of mRNA in single cells and modelling of RT-qPCR induced noise. BMC Mol Biol.

[30] Murphy J., Bustin S.A. (2009). Reliability of real-time reverse-transcription PCR in clinical diagnostics: gold standard or substandard?. Expert Rev Mol Diagn.

[31] Baker S.C. (2005). The External RNA Controls Consortium: a progress report. Nat Methods.

[32] FDA (2005). Guidance for industry pharmacogenomic data submissions.

[33] EPA (2004). Quality assurance/quality control guidance for laboratories performing PCR analyses on environmental samples.

[34] CLSI (2006). Use of external RNA controls in gene expression assays.

[35] Lavagnini I., Magno F. (2007). A statistical overview on univariate calibration, inverse regression, and detection limits: application to gas chromatography/mass spectrometry technique. Mass Spectrom Rev.

[36] Andrade J.M., Andrade-Garda J.M. (2013). Classical linear regression by the least squares method. Basic chemometric techniques in atomic spectroscopy.

[37] CLSI (2003). CLSI document EP6-A: evaluation of the linearity of quantitative measurement procedures.

[38] CLSI (2004). CLSI document EP5-A2: evaluation of precision performance of quantitative measurement methods.

[39] Cuthbert D. (1975). Calibration designs for machines with carry-over and drift. J Qual Technol.

[40] Kubista M. (2010). When to use interplate calibrator. http://www.tataa.com/products-page/quality-assessment/tataa-interplate-calibrator/.

